# Icariin Promotes Survival, Proliferation, and Differentiation of Neural Stem Cells In Vitro and in a Rat Model of Alzheimer's Disease

**DOI:** 10.1155/2021/9974625

**Published:** 2021-06-23

**Authors:** Denglei Ma, Lihong Zhao, Li Zhang, Yali Li, Lan Zhang, Lin Li

**Affiliations:** ^1^Department of Pharmacy, Xuanwu Hospital of Capital Medical University, Beijing 100053, China; ^2^National Clinical Research Center for Geriatric Diseases, Beijing 100053, China; ^3^Beijing Engineering Research Center for Nervous System Drugs, Beijing 100053, China; ^4^Beijing Institute for Brain Disorders, Beijing 100053, China; ^5^Key Laboratory for Neurodegenerative Diseases of Ministry of Education, Beijing 100053, China

## Abstract

Alzheimer's disease (AD) involves the degeneration of cholinergic neurons in the basal forebrain. Neural stem cell (NSC) transplantation has emerged as a promising therapeutic approach for treating AD. Icariin (ICA) is the main active component in *Epimedium*, a traditional Chinese herb. The purpose of the present study was to investigate the effects and mechanisms of ICA on the proliferation and differentiation of NSCs in the basal forebrain of a fimbria-fornix transection (FFT) rat model. In the present study, ICA promoted the survival, proliferation, and migration of NSCs *in vitro*. In FFT rats, ICA promoted the proliferation and differentiation of NSCs into neurons and increased the number of cholinergic neurons in the MS and VDB of the basal forebrain. These results suggest that combination therapy of ICA oral administration and NSC transplantation may provide a new potential and effective approach for AD therapy.

## 1. Introduction

Alzheimer's disease (AD), the most common type of dementia, involves the degeneration of cholinergic neurons in the basal forebrain. Neural stem/progenitor cells are multipotent stem cells with self-renewal capabilities with the ability of differentiation into neurons and glia cells. Neural stem cell (NSC) transplantation has emerged as a promising therapeutic approach for treating neurodegenerative diseases, such as AD [1]. However, NSCs need continuous support by neurotrophic factors for long-term survival after differentiating into a specific neuronal type following transplantation [2]. In AD treatment using exogenous NSC transplantation, a critical step is to induce the differentiation of NSCs into specific neurons.

The focal points of NSC transplantation are to maintain the activity, proliferation, and directional differentiation of NSCs after transplantation. Growth factors such as brain-derived neurotrophic factor (BDNF), glial cell-derived neurotrophic factor (GDNF), and nerve growth factor (NGF) are reported to promote the differentiation of NSCs into neurons *in vitro* and *in vivo* [3–5]. The epidermal growth factor (EGF) and basic fibroblast growth factor (bFGF) induce proliferation of NSCs and the formation of cell clusters and neurospheres [6]. However, growth factors are uneasy to cross the blood-brain barrier and show short half-life [1, 7]. Finding new small molecules to replace growth factors that maintain the activity and proliferation of transplanted stem cells becomes one of the key problems in this area [1, 8].

Epimedium (yinyanghuo) is a widely used traditional Chinese medicine for thousands of years. Epimedium flavonoids (EF) are the main active ingredient extracted from Epimedium and have been reported to have neuroprotective and anti-inflammatory effects [9, 10]. In our previous study, we found that Epimedium flavonoids effectively promoted the proliferation and differentiation of neural stem cells *in vitro* [11]. Icariin (ICA) is the main active component in Epimedium flavonoids. In the recent years, icariin has been found to exhibit pharmacological effects on several diseases in the nervous system, including AD, cerebral ischemia, multiple sclerosis, and Parkinson's disease [12–17]. Our previous study showed that ICA promoted the proliferation of neural stem cells *in vitro* [18]. However, the effects of ICA on NSC transplantation in AD models has not been investigated yet.

The fimbria-fornix transection (FFT) rat model has served as an AD animal model because FFT injures the projections of cholinergic nerve fibers in the septum to the hippocampus and the cortex, so as to induce cholinergic system damage and learning and memory impairment [19, 20]. In the present study, we first investigated the effects of ICA on the survival, proliferation, migration, and differentiation of NSCs *in vitro*. Then, we used FFT rats as an AD model and investigated the effects of ICA oral administration on the proliferation and differentiation of NSCs in the basal forebrain of the FFT rat model after NSC transplantation.

## 2. Materials and Methods

### 2.1. Drug and Materials

Icariin (ICA) (purity > 98% by high-performance liquid chromatography) was purchased from Shaanxi Scidoor Hi-tech Biology Co. Ltd. (Xi'an, China). Growth medium was composed of serum-free conditioned Dulbecco's modified Eagle's medium/Ham's F-12 medium (DMEM/F12, Gibco) with 20 ng/ml EGF and 10 ng/ml bFGF (Gibco, USA). NSC proliferation medium was composed of DMEM/F12 and B27 (2%) supplemented with 20 ng/ml EGF + 20 ng/ml bFGF. Basic medium was composed of DMEM/F12 and B27 (2%). NSC differentiation medium was composed of DMEM/F12, B27 (2%), GlutMAX (1%), and FBS (1%) (Gibco, USA). All cell plates or covered glasses were precoated with poly-D-lysine (Sigma, USA).

### 2.2. Preparation and Identification of NSCs

Single cells were isolated from telencephalon of embryonic day 14 rats and plated onto a cell culture flask in the growth medium serum-free conditioned medium DMEM/F12 containing 20 ng/ml EGF and 10 ng/ml bFGF. Cultures were maintained at 37°C in a humidified atmosphere of 95% air/5% CO_2_. After culture for 7 days, the proliferating cells formed the floating aggregates of a spherical shape, so-called neurospheres. Neurospheres were collected and mechanically dissociated to a single-cell suspension and replated in the NSC proliferation medium to generate secondary neurospheres.

After culture for 4 days in NSC proliferation medium, the secondary neurospheres were replated in 24-well plates after resuspension. 24 h later, the neurospheres were labeled with 10 *μ*M 5-bromo-2′-deoxyuridine (BrdU) (Sigma-Aldrich, USA). 48 hours later, cells were harvest and BrdU staining and immunofluorescence staining of Nestin were applied to identify the NSCs.

The secondary neurospheres, cultured for 7 days, were replated in NSC differentiation medium. After 7 days of differentiation, immunofluorescent staining for markers of neuron (*β*-III-tubulin) and astrocyte (glial fibrillary acidic protein (GFAP)) were conducted to identify the differentiation ability of neurospheres.

### 2.3. Cell Survival Assay Using CCK8

To study the effects of ICA on NSC survival, dissociated single cells from primary neurospheres (2 × 10^5^ cells/ml) were resuspended in NSC proliferation medium and cultured for 24 h. The medium was then replaced with basic medium containing different concentrations of ICA (final concentrations 0.1, 1, 10, and 20 *μ*M) and incubated with cells for 48 h. Cell survival assay using CCK8 was conducted as previously reported [18].

### 2.4. Cell Proliferation Assay Using BrdU

To study the effects of ICA on NSC proliferation, the secondary neurospheres cultured for 4 days were replated in NSC proliferation medium and cultured in a 48-well plate for 24 h. The medium was replaced with basic medium containing different concentrations of ICA (final concentrations 0.1, 1, 10, and 20 *μ*M) or NSC proliferation medium (containing EGF and bFGF, as positive control) and incubated with cells for 48 h. 10 *μ*M BrdU was added and cultured for 24 h. The number of BrdU-positive cells was then determined by BrdU staining.

### 2.5. Quantification of the Cell Migration Distance

To examine the effects of ICA on neurosphere differentiation, primary neurospheres were uniformly seeded on the bottoms of a 35 mm dish. Neurospheres were plated at a low density (10–15 neurospheres per dish) to ensure a large distance between individual spheres and incubated with basic DMEM/F12 medium without bFGF and EGF. The cultures were then treated with ICA (0, 0.1, 1.0, 10, and 20 *μ*M). At 1, 3, 7, and 14 days, quantification of the process growth was evaluated at the end-to-end distance of the extensive processes (NIS-Elements BR software). The lengths of 10–15 longest processes were estimated from the edge of the neurospheres or cell bodies to the tip of the processes.

### 2.6. Differentiation of NSCs

Dissociated single cells (2 × 10^4^ cells/well) from secondary neurospheres were plated at 24-well plates in NSC differentiation medium with ICA (0.1, 1.0, 10, and 20 *μ*M) or without ICA. Incubation was terminated at 7 days after plating, and the number of Tuj1 or GFAP and Hoechst-positive cells were counted after immunofluorescence staining.

### 2.7. Animals and FFT Surgery

Male Sprague–Dawley rats (250–270 g body weight) were obtained from Beijing Vital River Laboratory Animal Technology Co. Ltd., Beijing, China. Rats underwent unilateral fimbria-fornix transection (FFT) (*n* = 18) or sham operation (*n* = 6). Unilateral FFT was performed using a wire knife attached to a stereotaxic frame (SN-2 type, Narishige Ltd., Japan) after anesthesia as previously described [21]. Sham surgery animals were operated by opening the skull only. The efficiency of the axotomy was proven by Nissl's staining [22]. The rats were placed on a heating pad during recovery from anesthesia to maintain the body temperature after surgery. All animal care and experimental procedures were approved by the Bioethics Committee of Xuanwu Hospital of Capital Medical University.

### 2.8. NSC Transplantation and ICA Treatment

**Figure 1:**
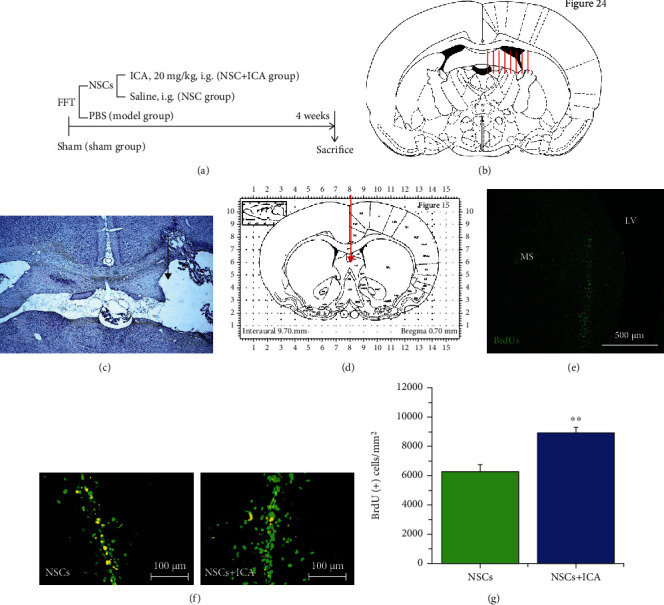
Verifications on the locations of fimbria-fornix transection (FFT) surgery and NSC transplantation *in vivo*. (a) Flow chart of experimental schedule *in vivo*. (b) Unilateral FFT surgery was conducted on rats (red arrow indicates the locations of FFT surgery), and (c) Nissl staining was conducted to verify the unilateral FFT-induced brain injury. After FFT surgery, (d) NSCs (2.5 × 10^4^/*μ*l × 4 *μ*l) were injected into the basal forebrain of rats: AP: +0.6 mm, LL+0.6 mm, and DV-5.5 mm from the bregma (red arrow). 28 days later, (e) the BrdU immunostaining indicated the survived NSCs near the needle track in the medial septum (MS) of the basal forebrain. (f) Representative images of BrdU-labeled transplanted NSCs in the brain of FFT rats. Scale bar = 100 *μ*m. (g) Quantitative analysis of the number of BrdU-positive cells in the NSC transplantation group and NSC transplantation-combined ICA treatment (NSC + ICA) group of FFT rats. Data are expressed as the mean ± SEM, *n* = 3. ^∗∗^*P* < 0.01, the NSC + ICA group*vs* the NSC transplantation group. LV: lateral ventricles.

Rats were randomly divided into 4 groups (Figure 1(a)): sham operation group, model group, NSC transplantation group, and NSC transplantation combined 20 mg/kg ICA (NSC + ICA) group. NSCs were isolated and cultured as described above, and single cells from secondary neurospheres were labeled with 10 *μ*M BrdU. NSCs (2.5 × 10^4^/*μ*l × 4 *μ*l) were stereotaxically injected into the basal forebrain of rats (AP + 0.6 mm, LL + 0.6 mm, and DV − 5.5 mm from the bregma) after FFT surgery. Intragastric administration of ICA (20 mg/kg/day) or vehicle was started 3 h after FFT surgery and continued for 28 days.

### 2.9. Immunofluorescence Staining and BrdU Staining

For immunofluorescence analysis of the cultured neurosphere or cells, cells were fixed in ice-cold 4% paraformaldehyde for 15 minutes and then washed three times in 0.01 M phosphate-buffered saline (PBS) (pH 7.4). For immunofluorescence analysis of the brain, brain slices were obtained following the procedures in the previous article [22]. After blocking using serum, brain slices or cultured cells were incubated with the primary antibodies at 4°C overnight. The antibodies used in this study are listed as follows: anti-Nestin (1 : 200; SAB4200347, Sigma-Aldrich, USA), anti-*β*-III-tubulin (Tuj1; 1 : 500; MAB1637, Sigma-Aldrich, USA), anti-NF-200 (1 : 200; N0142, Sigma-Aldrich, USA), anti-ChAT (1 : 200; A01192-4, Boster, Wuhan, China), and anti-GFAP (1 : 200; sc-33673, Santa Cruz, USA).The sections were then incubated with Alexa Fluor second antibody (1 : 200; Thermo Fisher Scientific, USA) for 2 h and counterstained by Hoechst 33342 (C1022, Beyotime, Shanghai, China) for 20 mins. The sections were then cover slipped with mounting medium (ZSGB-Bio, Beijing, China).

The proliferative activity of NSCs and verification of NSCs after transplantation were investigated by BrdU labeling and staining. Cultured cells or brain sections were treated with 2 M HCl, at 37°C for 30 mins to denature DNA, and added in 0.1 M borate buffer (pH 8.5) for 10 mins. Sections were then incubated with anti-BrdU antibody (1 : 200; B2531, Sigma-Aldrich, USA) and Alexa Fluor second antibody. Fluorescent signals were visualized by the confocal laser microscope system (TCS SP5, Leica, Germany).

### 2.10. Statistical Analysis

All data were analyzed using the software package SPSS 11.0 (SPSS Inc., Chicago, IL, USA). To compare significant differences between two groups, a two-tailed Student's *t*-test was used. One-way ANOVA analysis followed by *Dunnett's post hoc test* was used to determine statistical significance among more than two groups. Numerical data were provided as mean ± standard error of the mean (SEM), and a *P* value less than 0.05 was considered to be statistically significant.

## 3. Results

### 3.1. Identification of NSCs Isolated from Embryonic Day 14 Rats In Vitro

**Figure 2:**
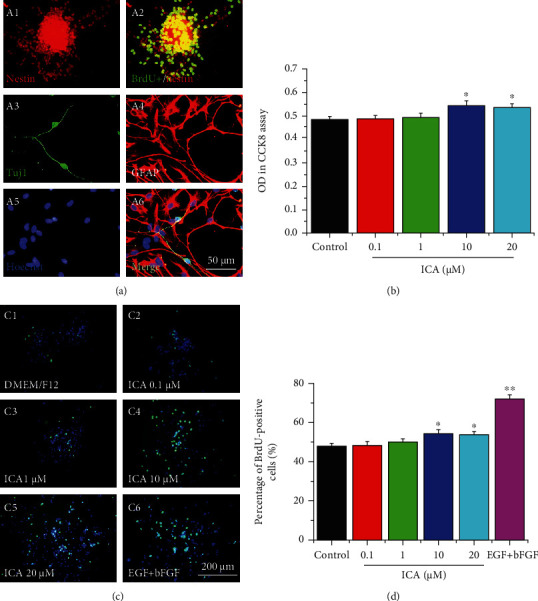
Identification of NSCs isolated from embryonic day 14 rats and beneficial effects of ICA on the survival and proliferation of NSCs *in vitro*. (a) Representative images of nestin-positive cells (A1, green) and BrdU-positive cells (A2, red) in a neurosphere. After differentiation, NSCs were immunoreactive to neuron marker Tuj1 (A3, green) and astrocyte marker GFAP (A4, red). Hoechst (A5) and Merge (A6). Scale bar = 50 *μ*m. (b) The optical density of NSCs treated with different concentrations of ICA as measured by cell-count kit-8 (CCK-8) assay, *n* = 6. (c) Immunostaining of BrdU-labelled NSCs under different proliferation mediums (C1: basic DMEM/F12 medium; C2-5: basic medium with ICA at 0.1, 1, 10, and 20 *μ*M; C6: basic medium with EGF 20 ng/ml and bEGF 20 ng/ml), scale bar = 200 *μ*m. (d) Quantitative analysis of the percentage of BrdU-positive cells. Data are expressed as the mean ± SEM, *n* = 25. ^∗^*P* < 0.05 and ^∗∗^*P* < 0.01, drug-treated groups *vs* the control group.

The results of immunofluorescent staining indicated that the neurospheres were immunopositive for nestin, which is the marker of NSCs (Figure 2(a), A1). BrdU labeling and immunofluorescent staining indicated that BrdU was incorporated into the majority of cells' nuclei of the neurospheres and showed positive staining (Figure 2(a), A2). Under differential conditions, some of the cells were immunoreactive to the neuron marker Tuj1 and some to the astrocyte marker GFAP (Figure 2(a), A3–A6). These results indicated that the cultured cells exhibited the characteristics of NSCs due to their self-renewal and multipotency and could be used to the transplantation study.

### 3.2. ICA Promoted the Survival and Proliferation of NSCs In Vitro

For observing the effects of ICA on NSC survival, dissociated cell from primary neurospheres were plated in 96-well plates and cultured for 48 h in the basic medium with different concentrations of ICA (0, 0.1, 1, 10, and 20 *μ*M). Results from CCK-8 cell activity assay indicated that 10 and 20 *μ*M ICA treatment promoted the survival of NSCs (*P* < 0.05, Figure 2(b)).

Secondary neurospheres were replated in with NSC proliferation medium (containing EGF and bFGF as positive control) or basic medium with different concentrations of ICA, and BrdU (10 *μ*M) was added to investigate the effects of ICA on cell proliferation. The percentage of BrdU-positive cells from Hoechst-positive cells showed that 10 and 20 *μ*M ICA treatment significantly promoted the proliferation of NSCs in the absence of EGF and bFGF (*P* < 0.05, Figures 2(c) and 2(d)).

### 3.3. Effects of ICA on the Migration and Differentiation of NSCs In Vitro

**Figure 3:**
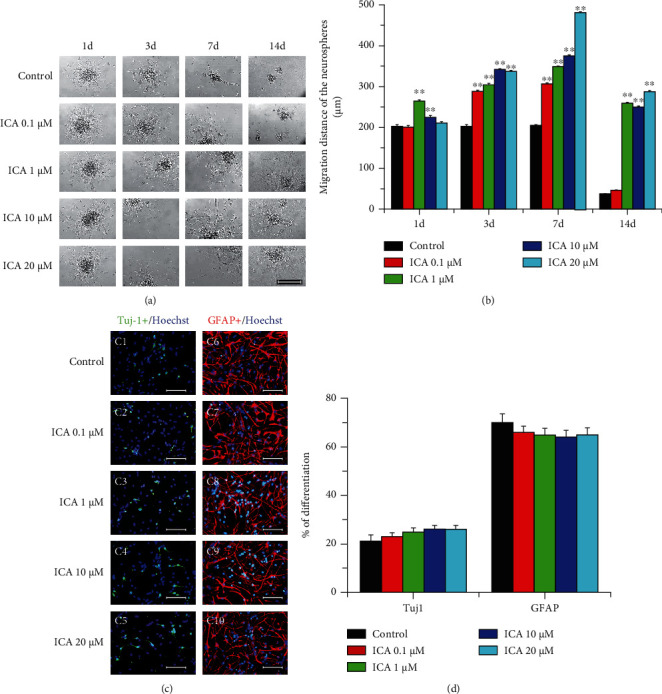
ICA promoted the migration ability and differentiation of NSCs *in vitro*. (a) Representative images of individual NSCs migrated from the neurospheres incubated in B27 medium without bFGF and EGF at 1, 3, 7, and 14 days. Scale bar = 200 *μ*m. (b) Quantitative analysis of the migration distance of the neurospheres at different time points after ICA incubation. Data are expressed as the mean ± SEM, *n* = 10. (c) Immunostaining of Tuj1 or GFAP to determine the effect of ICA on the differentiation of NSCs after seven days of incubation. Scale bar = 100 *μ*m. (d) Quantitative analysis of the percentage of Tuj1 or GFAP-positive cells to Hoechst-labeled NSCs. Data are expressed as the mean ± SEM, *n* = 25. ^∗∗^*P* < 0.01, drug-treated groups *vs* the control group.

To examine the effects of ICA on the NSC migration pattern from the neurosphere, neurospheres were seeded at a low density to ensure a large distance between individual spheres and incubated in basic medium without bFGF and EGF. Individual NSCs migrated rapidly away from the spheres, resulting in a rim of cells that formed a monolayer around the spheres, especially apparent on the periphery of the attached neurospheres. We selected 4 time points, i.e., 1, 3, 7, and 14 days, to examine the spheres' motility. ICA treatment promoted greater migration of NSCs than that of the control at all selected time points with a longer migration distance (*P* < 0.01; Figures 3(a) and 3(b)). On day 14, the majority of the cells died in the basic medium control group, whereas many single cells with long, straight, and slender processes migrated away from the core of neurospheres in 1, 10, and 20 *μ*M ICA groups (*P* < 0.01; Figures 3(a) and 3(b)).

To determine the effect of ICA on the differentiation of NSCs, single cells dissociated from neurospheres were reseeded and cultured in basic medium with ICA. Incubation was terminated at 7 days after plating and the number of Tuj1 or GFAP and the number of Hoechst-positive cells were counted. Immunofluorescent staining showed no difference between groups in the percentage of Tuj1 or GFAP-positive cells (Figures 3(c) and 3(d)).

### 3.4. ICA Promoted the Survival of NSCs after Transplantation into the FFT Rat Model

The unilateral FFT rat model was established as an animal model of AD [19, 20]. NSCs labelled with BrdU (2.5 × 10^4^/*μ*l ; 4 *μ*l) were stereotaxically injected into the basal forebrain of FFT rats. One group of FFT rats was treated with NSC transplantation combined with 20 mg/kg ICA, to observe the effects of ICA on NSCs *in vivo* (Figure 1(a)).

The location of the FFT surgery was chosen as shown in Figure 1(b) and verified by the Nissl staining (Figure 1(c)). And Immunofluorescent staining of BrdU-positive cells 28 days after FFT showed the transport site and the needle track, indicating the success of NSC transplantation (Figures 1(d) and 1(e)). We analyzed the number of BrdU-positive cells near the needle tracks of different groups. Compared with the NSC group, ICA treatment significantly increased the number of BrdU(+) cells near the needle tracks 28 days after NSC transplantation (*P* < 0.01; Figures 1(f) and 1(g)). These results indicated that ICA could increase the survival or proliferation of NSCs after transplantation *in vivo*.

### 3.5. ICA Promoted NSC Differentiation into Neurons and Astrocytes

**Figure 4:**
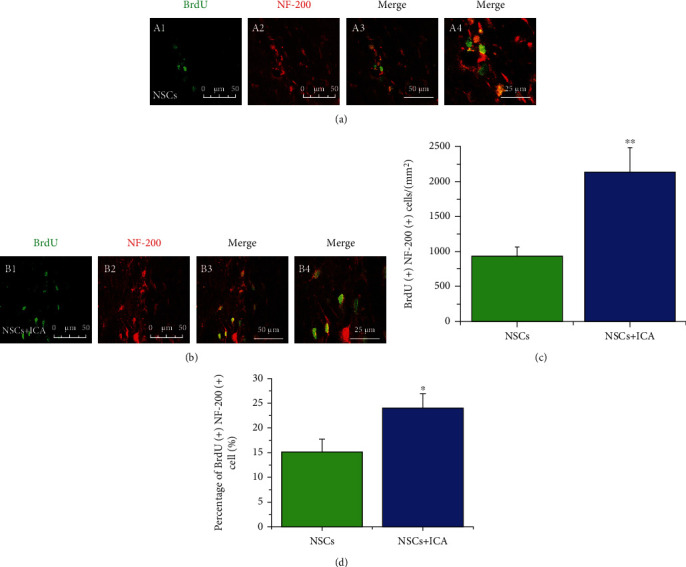
ICA treatment promotes transplanted NSCs differentiated into neurons in the FFT rat model. Double immunofluorescent staining of BrdU and NF-200 was applied to detect the number of neurons differentiated from NSCs of the NSC transplantation group (a) and NSC + ICA group (b). Scale bar = 50 *μ*m (A1–3, B1–3); 25 *μ*m (A4, B4). (c) Quantitative analysis of the number of NF-200(+)- and BrdU(+)-positive cells *in vivo*. (d) Quantitative analysis of the percentage of BrdU(+)/NF200(+) cells to BrdU(+) cells *in vivo*. Data are expressed as the mean ± SEM, *n* = 3. ^∗^*P* < 0.05 and ^∗∗^*P* < 0.01, the NSC + ICA group *vs* the NSC transplantation group.

NF-200 was used as the marker of mature neurons. Double immunofluorescent staining of BrdU and NF-200 was applied to detect the number of neurons differentiated from NSCs. Compared with vehicle-treated NSC group, ICA increased the number of BrdU/NF200-positive cells (*P* < 0.01; Figures 4(a)–4(c)) and the percentage of BrdU/NF200-positive cells (*P* < 0.05; Figure 4(d)). These results indicated that ICA treatment increased the number and percentage of NSCs differentiating into neurons.

**Figure 5:**
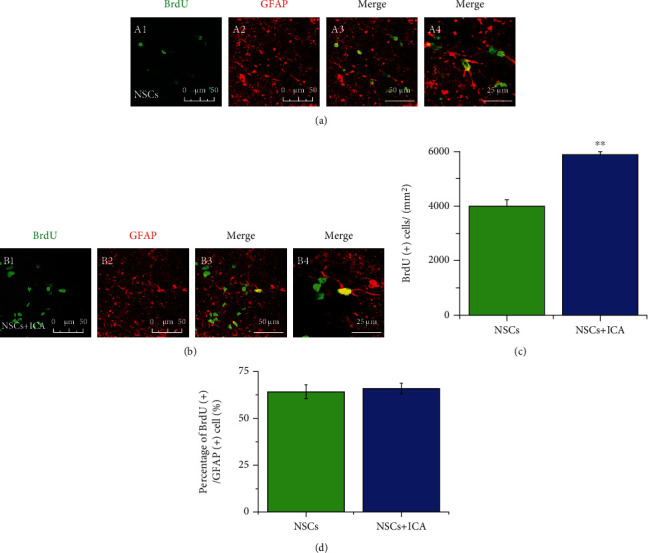
ICA treatment increased the total number of astrocytes differentiated from transplanted NSCs in the FFT rat model. Double immunofluorescent staining of BrdU and GFAP was applied to detect the number of astrocytes differentiated from NSCs of the (a) NSC transplantation group and (b) NSC + ICA group. Scale bar = 50 *μ*m (A1–3, B1–3); 25 *μ*m (A4, B4). (c) Quantitative analysis of the number of GFAP(+)- and BrdU(+)-positive cells *in vivo*. (d) Quantitative analysis of the percentage of BrdU(+)/GFAP(+) cells to BrdU(+) cells *in vivo*. Data are expressed as the mean ± SEM, *n* = 3. ^∗^*P* < 0.05 and ^∗∗^*P* < 0.01, the NSC + ICA group *vs* the NSC transplantation group.

GFAP was used as the marker of astrocytes. Double immunofluorescent staining of BrdU and GFAP was applied to detect the number of astrocytes differentiated from NSCs. Compared with vehicle-treated NSC group, ICA increased the number of BrdU/GFAP-positive cells (*P* < 0.01; Figure 5(a)–5(c)), with no effects on the percentage of BrdU/GFAP-positive cells (Figure 5(d)). These results indicated that ICA treatment increased the number of astrocytes differentiated from NSCs.

### 3.6. ICA Increased the Number of Cholinergic Neurons in the Basal Forebrain of FFT Rats

**Figure 6:**
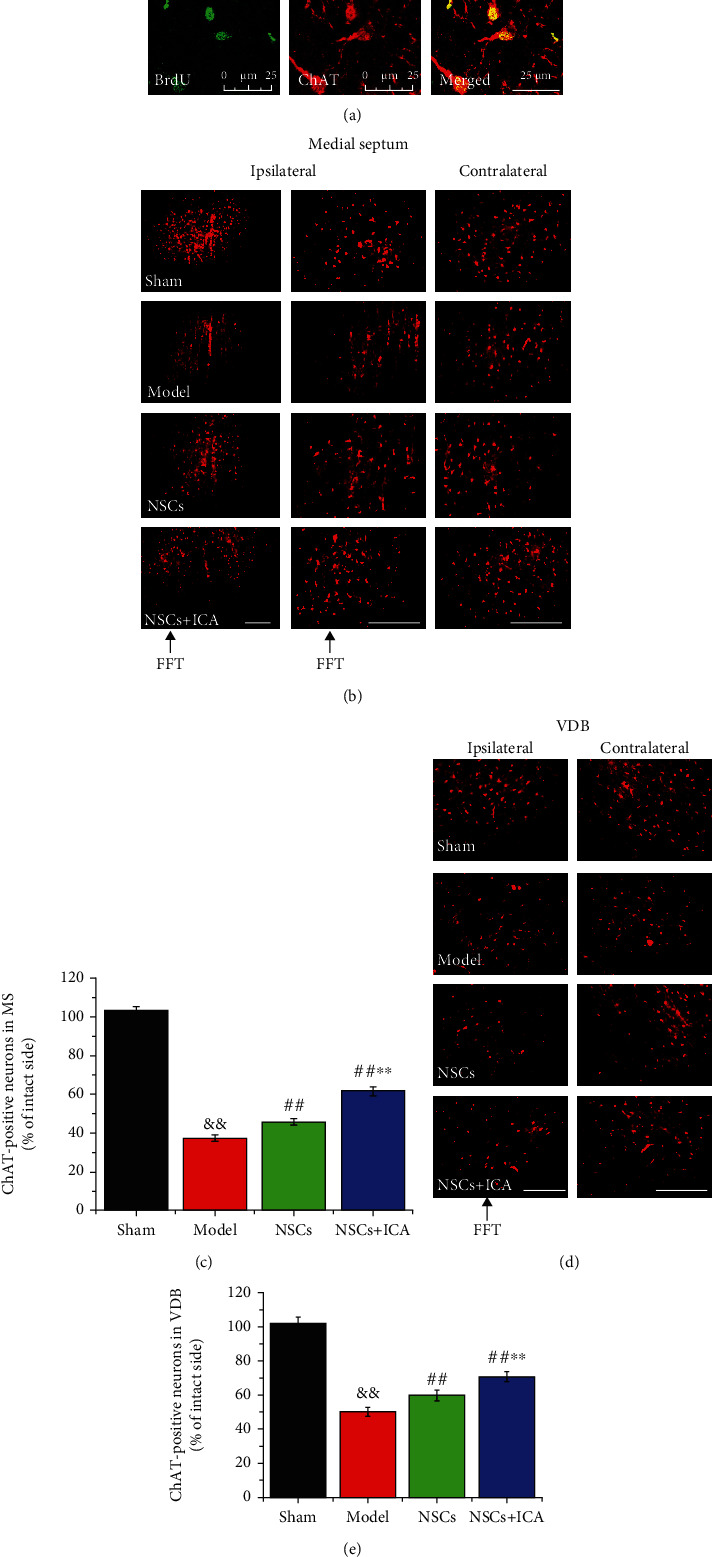
Combination of NSC transplantation and ICA treatment increased the total number of cholinergic neurons in the basal forebrain of FFT rats. (a) Immunofluorescent staining of BrdU (A1, green) and ChAT (A2, red) showed NSCs differentiated into cholinergic neurons *in vivo*. Scale bar = 25 *μ*m. (b) Immunofluorescent staining of ChAT in the medial septum (MS) of the ipsilateral and contralateral sides in FFT rats. Scale bar = 100 *μ*m. (c) Quantitative analysis of the ratio of ChAT(+) neurons in the ipsilateral side compared to contralateral side (intact side) in MS of FFT rats. (d) Immunofluorescent staining of ChAT in the vertical limb of the diagonal band (VDB) of the ipsilateral and contralateral sides in BF of FFT rats. Scale bar = 100 *μ*m. (e) Quantitative analysis of the ratio of ChAT(+) neurons in the ipsilateral side compared to the contralateral side (intact side) in VDB of FFT rats. Data are expressed as the mean ± SEM, *n* = 3. ^&&^*P* < 0.01, the FFT model group *vs* the sham group; ^##^*P* < 0.01, the NSC transplantation group *vs* the FFT model group; ^∗∗^*P* < 0.01, the NSC + ICA group *vs* the NSC transplantation group. ChAT: choline acetyltransferase (a marker of cholinergic neuron).

After FFT, the percentages of cholinergic neurons at the lesion side (ipsilateral) to the intact side (contralateral) in the medial septum (MS) and the vertical limb of the diagonal band (VDB) were significantly decreased (*P* < 0.01; Figure 6). In the NSC group and ICA-combined NSC therapy groups, the percentages of cholinergic neurons at the lesion side to the intact side in MS and VDB were significantly higher than those in the model group (*P* < 0.01; Figure 6). In the ICA-combined NSC group, there were more cholinergic neurons in MS and VDB compared with the NSC group (*P* < 0.01; Figure 6). These results indicated that NSC transplantation ameliorated the loss of cholinergic neurons induced by FFT in the MS and VDB of the basal forebrain and ICA treatment combined with NSC transplantation had better effects on the survival of cholinergic neurons.

## 4. Discussion

AD is a neurodegenerative disease with very complex pathophysiology. Extracellular senile plaques consisting of amyloid-*β* (A*β*) and intracellular neurofibrillary tangles (NFTs) composed of hyperphosphorylated tau lead to the neuron degeneration and synaptic loss, especially the loss of cholinergic neurons in the basal forebrain [22, 23]. NSC transplantation provides a promising strategy for the treatment of neurodegenerative disorders. However, little is known on how long this phenomenon can persist after NSC transplantation in AD [1]. A combination of NSC transplantation alongside administrating effective medication might be a more effective therapy for AD [8].

In the present study, we successfully isolated, cultured, and identified the NSCs from telencephalon of embryonic day 14 rats (Figure 2(a)). NSCs can self-renew and differentiate into neurons and glial cells. Then, we investigated the effects of ICA on the survival, proliferation, and differentiation of NSCs. Our studies demonstrated that ICA effectively promoted the survival of NSCs as single cells *in vitro* without showing toxic effects at the concentration of 0.1 ~ 20 *μ*M (Figure 2(b)). ICA increased the number of BrdU-labeled cells in cultured neurospheres (Figures 2(c) and 2(d)), and intragastric administration of ICA also increased the BrdU-labeled cells near the NSC transplantation site in the basal forebrain of FFT rats (Figures 1(f) and 1(g)). These results demonstrated that ICA promoted NSC proliferation directly *in vitro* and in the FFT rat model after NSC transplantation.

NSC proliferation and migration and neural differentiation are key processes for neurogenesis and regeneration in CNS [24, 25]. In the present study, we detected the effects of ICA on the migration and differentiation of NSCs. The results showed that ICA treatment promoted greater migration of NSCs with longer migration distances at 1, 3, 7, and 14 days after neurospheres were seeded. Furthermore, NSCs can survive longer (at least 14 days after seeding) with ICA treatment in basic medium without bFGF and EGF (Figures 3(a) and 3(b)). These results indicated that ICA promoted the migration and survival of NSCs, which may provide more capacity of neurogenesis and regeneration in the brain after NSC transplantation.

NSCs have the potential to differentiate into neurons and glial cells under certain conditions, which are the source for functional regeneration of damaged CNS after NSC transplantation [25]. However, NSCs show more tendency of differentiation into astrocytes other than neurons, with only a minor proportion differentiated into neurons *in vitro* and *in vivo* [26]. In the present study, ICA treatment increased the number of neurons and astrocytes differentiated from NSCs in the brain of FFT rats. Moreover, ICA increased the percentage of NSCs' differentiation into neurons (Figures 4 and 5). NSCs rarely differentiate into neurons because of the adverse microenvironment present in CNS damage [27]. So, we infer that ICA treatment may benefit the differentiation of neurons by improving the microenvironment in the brain of FFT rats.

Neurotrophic factors such as BDNF and NGF have been reported to improve the microenvironment and promote the differentiation of NSCs into neurons [3, 5]. The interventions that increase neurotrophic factors can promote the differentiation of NSCs [27, 28]. Our previous study showed that Epimedium flavonoids increased the expression levels of BDNF and neuregulin-1 in the hippocampus of a chronic cerebral hypoperfusion rat model [9, 29]. ICA is the main active component in Epimedium flavonoids. We also found that ICA elevated the number of NeuN/NGF-positive cells in the frontal cortex of a demyelination mouse model [15]. Recently, ICA has been reported to sustain the proliferation and differentiation of A*β*_25-35_-treated hippocampal neural stem cells via the BDNF-TrkB-ERK/Akt signaling pathway [16]. In animal models of depression and traumatic brain injury, ICA was reported to increase the expression of BDNF and improve functional behaviors of the disease models [30, 31]. Therefore, we conjecture that ICA may increase neurotrophic factors which improve the microenvironment and promote the differentiation of NSCs into neurons.

The massive loss of cholinergic neurons in the basal forebrain is one of the major pathological changes in AD patients [23]. The fimbria-fornix constitutes a major afferent and efferent fiber tract connecting the hippocampus with the diencephalon, forebrain, striatum, and prefrontal cortex [32]. FFT deprives the hippocampus of its major cholinergic input and, furthermore, disrupts substantial parts of the output from the hippocampal formation [19, 20]. In the present study, we applied the FFT rat model to investigate the effects of ICA on transplanted NSCs *in vivo*. The results showed that the number of cholinergic neurons in MS and VDB of the basal forebrain was significantly decreased in the FFT model group. The combination therapy of daily ICA oral administration and NSC transplantation was applied on the rat model after FFT surgery. Although NSC transplantation alone increased the number of cholinergic neurons in MS and VDB of the basal forebrain, the combination therapy showed better effects (Figure 6). Loss of cholinergic neurons in the basal forebrain is correlated with the cognitive decline of AD patients [24, 33]. It has been reported that transplantation of cholinergic neurons improves the cognitive ability in AD models [1, 34, 35]. So, we speculate that combination therapy of ICA oral administration and NSC transplantation may improve the cognitive function in AD.

## 5. Conclusion

In the present study, we found that ICA promoted the survival, proliferation, and migration of NSCs *in vitro*. In FFT rats, ICA promoted the proliferation and differentiation of NSCs into neurons and increased the number of cholinergic neurons in the MS and VDB of the basal forebrain. These results suggest that combination therapy of ICA oral administration and NSC transplantation may provide a new potential and effective approach for AD therapy.

## Data Availability

All data used to support the findings of this study are available from the corresponding author upon reasonable request.
